# Evaluation of anesthesiologists’ knowledge about occupational health: Pilot study

**DOI:** 10.1186/s12871-018-0661-y

**Published:** 2018-12-19

**Authors:** Daniel Dongiu Kim, Aldemar Kimura Jr, Dayanne Karla Lopes Pontes, Maycon Luiz Silva Oliveira, Debora Oliveira Cumino

**Affiliations:** Department of Anesthesiology, Irmandade da Santa Casa de Misericordia de Sao Paulo, Rua Dr. Cesario Motta Jr, 112, Sao Paulo, SP Brazil

**Keywords:** Occupational health, Anesthesiology, Occupational risk, Risk management, Safety management

## Abstract

**Background:**

An anesthesiologists’ work presents with numerous occupational risks owing to the large amount of time spent inside the operating room where constant noise, anesthetic vapors, ionizing radiation, infectious agents, and psychological stress are present. Herein, we evaluated anesthesiologists’ knowledge about occupational health.

**Methods:**

A cross-sectional study was conducted to assess 158 anesthesiologists from a tertiary hospital on their knowledge about occupational health using a structured questionnaire.

**Results:**

The survey revealed a lack of knowledge on the forms of prevention of occupational accidents (74.6% did not know how to act in case of a fire during surgery, 56% failed to identify the post-anesthesia care unit as the place with the highest contamination by inhalation anesthetics, and 42.7% failed to identify all personal protective equipment) and a surprisingly high rate of lack of observance of preventive measures (30.3% washed their hands before touching every patient, 52.5% did not use gloves during intravenous access, and 88.6% used protective equipment against ionizing radiation).

**Conclusions:**

Despite improvements in safety standards in healthcare facilities, our research showed lack of knowledge about major topics on occupational health by physicians. Improving safety awareness is an important goal of training programs and continued medical education.

**Electronic supplementary material:**

The online version of this article (10.1186/s12871-018-0661-y) contains supplementary material, which is available to authorized users.

## Background

Studies on occupational health focus on the interaction between work and health-disease processes to reduce inherent hazardous conditions involving physical, psychological, and social risks at work places, improve employee performance, and preserve their capacity for work [1–3].

Healthcare workers are at a disadvantage because, historically, they are not considered to be at a high risk for occupational accidents and diseases. However, studies on the health and disease processes of these workers revealed alarming data on the exposure of these professionals to risk factors affecting their physical and mental integrity [4, 5]. Additionally, the increasing demand for healthcare services worldwide have weakened labor relations further, particularly in terms of worker training and supervision [6].

Anesthesiology is associated with risks because the professional practice mainly occurs within the operating room, where there is continuous noise pollution, chemical fumes, ionizing radiation, infectious agents, and high psychological stress level exposures. With the increasingly precarious work relationship associated with the lack of knowledge of occupational hazards, anesthesiologists become vulnerable; therefore, knowledge of occupational hazards and means of prevention are important for safeguarding workers’ health [7, 8].

In our literature review, we found studies that outlined the profile of anesthesiologists and their quality of life [9, 10] as well as research evaluating physical and psychological consequences of exposure to risks inherent to working in the operating room environment [11, 12]. However, we found no studies on the anesthesiologist’s knowledge of risks involved in professional practice. Therefore, we investigated the behavior of anesthesiologists when faced with various risks involved in their work environment to promote educational strategies targeting the prevention of occupational risks.

The present study is a pilot study aimed to describe the knowledge of anesthesiologists working in a tertiary university hospital regarding occupational risks in the surgical theater environment, personal protection equipment use, infection transmission risk, environmental exposure risk, and conduct during critical events and also determine whether the amount of clinical experience influences the knowledge of occupational risks and the adoption of precautionary measures.

## Methods

After approval from the institutional review board, we performed an active search for anesthesiology-related personnel (residents and staff anesthesiologists) at the institution and asked them to voluntarily and anonymously respond to a structured questionnaire after they had signed the informed consent form [Additional file 1].

Data obtained after excluding incomplete questionnaires were encoded and compiled for statistical analysis using version 3.00 of InStat program (GraphPad Software Inc., La Jolla, CA, USA). The Fisher’s exact test was used for analyzing the association between categorical variables. To check whether our non-significant results were due to a lack of statistical power post hoc power analyses using GPower software [13] was conducted with power (1 - β) set at 0.80 and α = 0.05 two-tailed.

Group G_1_ comprising anesthesiologists with < 10 years of experience and group G_2_ comprising those with > 10 years of experience were created for stratification and comparison according to the amount of clinical experience.

## Results

A total of 160 anesthesiologists participated in the study, and 158 questionnaires were valid (two questionnaires were incomplete and were excluded from statistical analysis). These 158 participants included 89 anesthesiology residents (56.3%), 11 anesthesiologists with up to 5 years of professional experience (7.0%), 24 anesthesiologists with 5–10 years of professional experience (15.2%), 17 anesthesiologists with 10–20 years of experience (10.8%), and 17 anesthesiologists with > 20 years of experience (10.8%). On stratification by amount of experience, group G_1_ included 124 interviewees and group G_2_ included 34 interviewees.

On overall evaluation of the questionnaire responses, four questions had > 50% correct responses (personal protection equipment necessary for patients under contact isolation precautions, conduct following needlestick and sharp injuries, personal protection equipment necessary for patients under droplet isolation precautions, and checking of the electric scalpel return electrode), four questions had 35–50% correct responses (procedure glove use during peripheral venous access puncture, the infectious/contagious disease with the highest occupational transmission risk, identification of the area of the operating room with the highest level of contamination by inhalation anesthetics, and which personal protection equipment is required for protection against ionizing radiation), and three questions had < 35% correct responses.

On stratification by amount of experience, there was no statistical difference between groups G_1_ and G_2_ for questions about personal protection equipment for protection against ionizing radiation, conduct in case of fire in the operating room, which personal protection equipment should be used by patients under protection against droplets, conduct after needlestick or sharp injury, and area of the operating room with the highest level of contamination by inhalation anesthetics. Group G_1_ had a higher proportion of correct responses than that of group G_2_, with statistical significance for questions about disposable glove use during peripheral venous access puncture, and contagious/infectious disease with the highest occupational transmission risk. Group G_2_ had a higher proportion of correct responses than that of group G_1_, with statistical significance for questions about handwashing before contact with patients, recapping of needles after use, checking of the electric scalpel return electrode, and personal protection equipment required for patients under contact isolation precautions.

Further statistical analysis shows that the absence of relationship between groups G1 and G2 for questions about personal protection equipment for protection against ionizing radiation, which personal protection equipment should be used by patients under protection against droplets, conduct after needlestick or sharp injury, and area of the operating room with the highest level of contamination by inhalation anesthetics may have occurred due to small sample size. With power (1-β) set at 0.8 the samples would have to be increased to 2868, 382, 226 and 226, respectively in order for group differences to reach statistical significance (α = 0.05); Table 1.

**Table 1:** Comparison between groups G_1_ and G_2_ for correct responses, statistical power (1-β) and intergroup association test [Additional file 1]

Questionnaire item	Correct responses (group, statistical power, and statistical test)			
	Group G_1_	Group G_2_	1-β	p
Appropriate protection against ionizing radiation	59	18	0.061	0.4311
	47.6%	52.9%		
Conduct in case of fire in the operating room	35	5	0.986	0.1240
	28.2%	14.7%		
PPE for droplet isolation precautions	100	23	0.332	0.2330
	80.6%	67.6%		
Area with the highest level of contamination by inhalatory agents	60	10	0.463	0.0610
	48.4%	29.4%		
Conduct after needle stick or sharp injury	89	18	0.502	0.0610
	71.8%	52.9%		
Agent with the highest contamination risk after an accident	57	8	0.645	0.0194
	46.0%	23.5%		
Disposable glove use during venous access	67	9	0.792	0.006
	54.0%	26.5%		
PPE for contact isolation precautions	63	27	0.857	0.003
	50.8%	79.4%		
Handwashing before contact with a patient	27	21	0.989	< 0.001
	21.8%	61.8%		
Recapping of a hypodermic needle after use	1	6	0.942	< 0.001
	0.8%	17.6%		
Checking the return electrode of the electric scalpel	18	17	0.982	< 0.001
	14.5%	50.0%		

## Discussion

There are few publications about occupational health for anesthesiologists and the majority is related with sanitary regulations and recommendations by administrative agencies [14, 15]. Measuring previous knowledge is an important tool for further educational activities and our research found a considerable heterogeneity in the knowledge of occupational hazards and their prevention.

Biological materials are one of the main occupational hazards for healthcare professionals. Accidents involving blood and other body fluids are the most frequently reported exposures [1, 16, 17]. Although there is joint effort by entities to improve the recording of and response to accidents with biological materials, there are no reliable global statistics available to date owing to difficulties in aggregating data [18].

Although both health legislation and medical literature recommend handwashing and glove use before contact with patients [16, 19], since wearing gloves are not substitute of hand hygiene due to glove leakage and self-contamination during removal. Research showed that up to 53% of gloves presented leakages post-use and up to 34% of healthcare worked presented positive cultures after gloves removal [20, 21]. Our study demonstrated a controversial behavior with more experienced professionals disclosing using disposable gloves less often for procedures but washing hands before each patient encounter more consistently than less experienced anesthesiologists. This finding is similar to a previous study showing an inversely proportional relationship between gloves use and hand hygiene [22].

Needlestick and sharp injuries present high risk owing to the potential of pathogen transmission. Infectious agents with the highest transmission risk after percutaneous exposure are in the following order: hepatitis B, hepatitis C, and HIV [17, 18]; this was correctly identified by 41.1% respondents in our study. Lack of awareness of the main infectious agent is concerning because it diminishes the adherence of professionals to hepatitis B vaccination, as evidenced by studies quantifying the low prevalence of serum anti-HBs in anesthesia-related personnel [23, 24]. Nevertheless, a systematic review demonstrated that needle injury is most likely to occur during syringe preparation and recapping [25], and in our research, needles had recapped more often for less experienced professionals.

In recent years the outbreak of arbovirus infections in Latin America raised the concern of occupational contamination especially after the association between Zika virus infection during pregnancy and microcephalia. There are several studies showing transmission of Dengue fever, Chikungunya and Zika virus through blood transfusion by asymptomatic donors or during incubation period and it is reasonable that needlestick and sharp injuries may carry a risk of transmission [26–28]. Also several healthcare facilities found vectors mosquitoes in their premises increasing the chances of healthcare workers arbovirus infection through classical form of transmission [29].

Although there is no evidence of direct cytotoxicity of inhaled anesthetic agents that would determine an increase in the incidence of diseases, malformations, or abortion in exposed professionals, these agents have genotoxic potential [11, 12]. In the operating room, the post anesthesia care unit is considered the area with the highest contamination secondary to multiple patients exhaling volatile anesthetics while in recovery from anesthesia. New research shows that adequate scavenging system can reduce post anesthesia care unit contamination to safe levels [30].

The development of minimally invasive procedures requiring fluoroscopy has increased the risk of occupational ionizing radiation exposure. To prevent stochastic effects of exposure, wearing lead-based garments and maintaining an appropriate distance from the fluoroscopic source are recommended [31].

Another concerning phenomenon is high flammability of the modern operating room owing to three main fire hazards: fuel (alcohol-based solutions and other solvents), combustion agent (oxygen in high concentrations), and energy (electric scalpel and lasers). Recent continued medical education programs are increasing awareness of fire risk during surgery. Anesthesiologists can prevent this catastrophic event by reducing the combustion agent during a surgical procedure [32, 33].

Additionally, the return electrode of an electric scalpel must be checked because it is an energy source that can cause burns and even fires. All modern electric scalpels feature grounding isolation, thereby providing greater safety during use, but they are not failure-free [34]. In our research, the more experienced professionals checked the position of the return electrode of the electric scalpel more often because many of them witnessed the advent of electrosurgery and the problems associated in the beginning of this technology [35].

Despite limitations of disproportionate sample size between G_1_ and G_2_ of this study, we detected interesting differences in conduct regarding occupational hazards according to the amount of clinical experience. Less experienced professionals are more aware of infectious diseases potentially transmitted by occupational accidents and wear gloves more often but also present a high risk behavior by recapping needles. The more experienced anesthesiologists have witnessed outbreaks of many infectious diseases and the development of new surgical technologies, and this is reflected by their behavior such as checking the position of the return electrode of the electric scalpel, consistent use of personal protective equipment, and lower rates of needle recapping.

This study has several limitations. This research was conducted in only one hospital and the results presented possibly are affected by institutional culture and may not be generalized. The paucity of research in this field grant further research especially in educational strategies to reduce the gap between knowledge and routine practice, particularly for training anesthesiologists. The future specialists will shape the medical practice in coming years for a healthier workplace for healthcare professionals, [36].

## Conclusions

Despite the existence of specific legislations and extensive medical literature, the knowledge of anesthesiologists interviewed in this study about occupational health is below the expected levels [1, 3].

In the present study, there was a difference regarding occupational hazards knowledge according to the anesthesiologists’ amount of clinical experience. Because professionals with more clinical experience have witnessed the advent of electrosurgery, development of less flammable inhaled anesthetics, and several epidemics caused by infectious/contagious agents, this has shaped the occupational safety knowledge in this group.

This finding reveals the importance of awareness and adherence to measures that effectively reduce occupational hazards. Increasing the knowledge of occupational hazards and corresponding prophylactic measures can potentially reduce the incidence and consequences of occupational diseases.

## Additional file

Additional file 1: Translated questionnaire applied for the research subjects. (DOCX 14 kb)

## Abbreviations

Anti-HBs: Anti-Hepatitis B surface antibody
Group G_1_: Anesthesiologists with < 10 years of clinical experience
Group G_2_: Anesthesiologists with > 10 years of clinical experience
HIV: Human Immunodeficiency Virus
PPE: Personal Protective Equipment
