# Supplementary material for: Evaluation of anesthesiologists’ knowledge about occupational health: Pilot study
Source: BMC Anesthesiol. 2018 Dec 19;18:193. doi: 10.1186/s12871-018-0661-y (PMC6300909; doi:10.1186/s12871-018-0661-y)
Supplement: Supplementary file 1 — Translated questionnaire applied for the research subjects. (DOCX 14 kb) [file 12871_2018_661_MOESM1_ESM.docx]

1. How many are you working as anesthesiologist? (in years)

| □ < 3 | □ < 5 | □ 5 to 10 | □ 10 to 15 | □ > 15 |
| --- | --- | --- | --- | --- |

1. How often do you use disposable glove use during venous access?

| □ Always | □ Very often | □ Often | □ Rarely | □ Never |
| --- | --- | --- | --- | --- |

1. Which PPE for contact isolation precautions do you use?

___________________________________________________________________________

1. How often do you handwashing before contact with a patient?

| □ Always | □ Very often | □ Often | □ Rarely | □ Never |
| --- | --- | --- | --- | --- |

1. Regarding bloodborne infectious diseases which agent poses the highest contamination risk after an accident?

| □ HIV | □ Hepatitis C | □ Hepatitis B | □ Hepatitis A | □ HPV |
| --- | --- | --- | --- | --- |

1. Which is the most important PPE that the anesthesiology should use when in contact with a patient with suspected active tuberculosis?

______________________________________________________________________________

1. How often do you recap a hypodermic needle after use during anesthesia?

| □ Always | □ Very often | □ Often | □ Rarely | □ Never |
| --- | --- | --- | --- | --- |

1. Which is the best first measure after needle stick or sharp injury?

______________________________________________________________________________

1. Which area present the highest level of contamination by inhalatory agents?

| □ Surgery room | □ PACU | □ Hallway | □ Holding area |
| --- | --- | --- | --- |

1. For protection against ionizing radiation you always use:

| □ Lead apron | □ Thyroid collar | □ Lead glasses | □ Exit the room |
| --- | --- | --- | --- |

1. The first measure in case of fire in the operating room:

______________________________________________________________________________

1. How often do you check the return electrode of the electric scalpel?

| □ Always | □ Very often | □ Often | □ Rarely | □ Never |
| --- | --- | --- | --- | --- |
